# A Toolbox for Site-Specific Labeling of RecQ Helicase With a Single Fluorophore Used in the Single-Molecule Assay

**DOI:** 10.3389/fmolb.2020.586450

**Published:** 2020-09-25

**Authors:** Fang-Yuan Teng, Zong-Zhe Jiang, Ling-Yun Huang, Man Guo, Feng Chen, Xi-Miao Hou, Xu-Guang Xi, Yong Xu

**Affiliations:** ^1^Experimental Medicine Center, The Affiliated Hospital of Southwest Medical University, Luzhou, China; ^2^State Key Laboratory of Crop Stress Biology for Arid Areas and College of Life Sciences, Northwest A&F University, Yangling, China; ^3^Department of Endocrinology and Metabolism, and Cardiovascular and Metabolic Diseases Key Laboratory of Luzhou, and Sichuan Clinical Research Center for Nephropathy, The Affiliated Hospital of Southwest Medical University, Luzhou, China; ^4^Academician (Expert) Workstation of Sichuan Province, The Affiliated Hospital of Southwest Medical University, Luzhou, China; ^5^LBPA, Ecole normale supérieure Paris-Saclay, Centre national de la recherche scientifique (CNRS), Université Paris Saclay, Cachan, France

**Keywords:** fluorescence, molecular interaction, molecular dynamic, DNA repair, single molecule, helicase, protein labeling

## Abstract

Fluorescently labeled proteins can improve the detection sensitivity and have been widely used in a variety of biological measurements. In single-molecule assays, site-specific labeling of proteins enables the visualization of molecular interactions, conformational changes in proteins, and enzymatic activity. In this study, based on a flexible linker in the *Escherichia coli* RecQ helicase, we established a scheme involving a combination of fluorophore labeling and sortase A ligation to allow site-specific labeling of the HRDC domain of RecQ with a single Cy5 fluorophore, without inletting extra fluorescent domain or peptide fragment. Using single-molecule fluorescence resonance energy transfer, we visualized that Cy5-labeled HRDC could directly interact with RecA domains and could bind to both the 3′ and 5′ ends of the overhang DNA dynamically *in vitro* for the first time. The present work not only reveals the functional mechanism of the HRDC domain, but also provides a feasible method for site-specific labeling of a domain with a single fluorophore used in single-molecule assays.

## Introduction

Fluorescently labeled proteins can help dissect the detailed molecular mechanisms and have become crucial experimental tools in various research fields, especially in flow cytometry, fluorescence microscopy, and enzymatic activity measurements (Li et al., 2015). There are a variety of fluorescent labeling methods and strategies that depend on particular applications, such as genetically encoded tags (Schlichthaerle et al., 2019), quantum dots (Abu-Thabit and Ratemi, 2020), and small organic fluorophores (Rosen and Francis, 2017). It can be performed in solution and in real time, with high time resolution and high sensitivity even at the single-molecule level (Toseland, 2013).

In single-molecule fluorescence resonance energy transfer (smFRET) assay, fluorescently labeled proteins can realize the visualization of molecular interactions, conformational changes in proteins, and enzymatic activity and the ability to detect individual proteins moving in real time (Aggarwal and Ha, 2016). In contrast to other applications, fluorescence labeling used in smFRET requires high precision because of its nanolevel sensitivity. Therefore, small organic fluorophores, such as NHS ester-activated cyanine, which can selectively couple with the N-terminus and lysine residues (Rosen and Francis, 2017), and maleimide-activated cyanine, which can selectively couple with cysteine residues (Friedman Ohana et al., 2019), have been mainly adopted to label the protein in smFRET because they are small and stable and do not perturb the real-time detection system. However, in the smFRET assay, it is important that the structure and activity of the protein will not be affected in fluorescence labeling process, and the fluorescence labeling site is specific, single, and homogeneous. Therefore, the specific strategy to label a small fluorophore to the indicated site plays a decisive role and becomes a key limiting factor in many smFRET detections.

**FIGURE 1:**
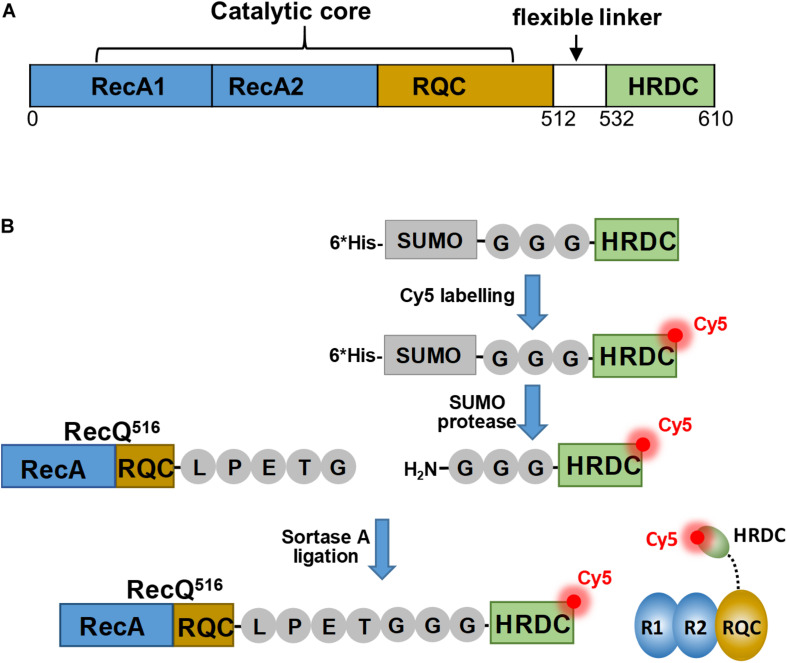
Construction strategy for sortase A–mediated site-specific labeling of HRDC with a single Cy5. **(A)** Schematic diagram of the domain structure of *E. coli* RecQ. **(B)** The scheme of specifically labeling HRDC with a single Cy5-maleimide fluorophore mediated by sortase A. The ligation was designed without changing the length of the non-functional flexible linker. R1, RecA domain 1; R2, RecA domain 2; and RQC, RecQ-C-terminal domain.

RecQ family helicases include *Escherichia coli* RecQ, and *Homo sapiens* RecQ1, BLM, WRN, RecQ4, and RecQ5β, which widely participate in multiple DNA metabolic processes and play key roles in maintaining genomic stability (Bell and Kowalczykowski, 2016; de Renty and Ellis, 2017). As a functional and structural prototype of RecQ family helicases, *E. coli* RecQ can help suppress illegitimate recombination, repair stalled replication forks at DNA damage sites, and unwind G-quadruplex DNA (Croteau et al., 2014; Mendoza et al., 2016). Two conserved domains have been identified among all RecQ family helicases: the helicase domain, which contains two RecA domains and is involved in ssDNA binding and NTP hydrolysis, and the RecQ-C-terminal (RQC) domain, which is primarily responsible for substrate recognition and DNA unwinding (Figure 1A; Bernstein et al., 2003; Swan et al., 2014; Newman et al., 2015).

Additionally, some RecQ family helicases, including *E. coli* RecQ, and *H. sapiens* BLM and WRN, contain an auxiliary domain: the helicase and RNaseD C-terminal (HRDC) domain, which is connected to the RQC domain by a flexible linker [more than 20 amino acids (aa)] (Morozov et al., 1997). The HRDC domain of BLM has been reported to possess markedly low ssDNA-binding affinity (*K*_*D*_ ∼100 μM) (Kim and Choi, 2010) and interacts directly with the two RecA domains, which may influence the mechanochemical coupling of the ATPase cycle (Swan et al., 2014; Newman et al., 2015). However, the HRDC of human WRN has been shown to lack DNA binding ability *in vitro*, and it is suggested that it may mediate protein–protein interactions (Kitano et al., 2007). Meanwhile, the isolated HRDC of *E. coli* RecQ could bind only to ssDNA by electrophoretic mobility shift assay analysis (Bernstein and Keck, 2005). Enzyme kinetics studies found that the HRDC domain could slow down ssDNA translocation and dsDNA unwinding processes (Kocsis et al., 2014; Harami et al., 2015). Therefore, HRDC from different proteins may have different functions, and direct visualization of the trajectory of HRDC during RecQ transaction with different DNA at the single-molecule level needs to be detected.

In this study, based on the flexible linker (∼22 aa) between the RQC and HRDC domain of *E. coli* RecQ helicase, we established a scheme involving a combination of fluorophore labeling and ligation by sortase A, which could fuse an LPXTG recognition motif to an N-terminal GGG motif, thus regenerating a native amide bond and a recombinant protein (Levary et al., 2011). Therefore, we replaced only six non-functional amino acids on the flexible linker without inletting extra fluorescent domain or peptide fragment, succeeded in site-specific labeling of the HRDC domain of *E. coli* RecQ with a single Cy5 fluorophore, and found that the enzymatic activity of RecQ after fluorophore-labeling had little impact on the smFRET assay. Meanwhile, we observed that the HRDC domain could directly interact with RecA domains and could bind to both the 3′ and 5′ ends of the overhang DNA repeatedly. The present work not only directly reveals the functional mechanism of the HRDC domain during *E. coli* RecQ transaction with different DNA during DNA repair or DNA recombination, but also provides a feasible method for site-specific labeling of a domain with a single fluorophore used in single-molecule assays.

## Materials and Methods

### Plasmid Construction, Protein Expression, and Purification

All DNA primers required making the protein constructs were purchased from Sangon Biotech (Shanghai, China), and the sequences were listed in Supplementary Table S1. All the indicated protein constructs were severally amplified from *E. coli* genome. The sumo-GGG-HRDC construct was obtained by overlap polymerase chain reaction (PCR) using the primer Sumo-F/Sumo-R, and HRDC-F/HRDC-R. RecQ^516^-LPETG construct was obtained by PCR using the primer RecQ^516^-LPETG-F and RecQ^516^-LPETG-R. After digestion by *Nde*I/*Xho*I, the indicated protein constructs were severally constructed into pET15b vector, and expressed in BL21 (DE3) induced by 0.3 mM IPTG at 18°C for 16 h. Then, the indicated recombinant protein was purified by Ni affinity chromatography (Shi et al., 2017). Briefly, after being harvested by centrifugation, each cell pellet was resuspended by ice-cold lysis buffer (10 mM imidazole and 500 mM NaCl in 20 mM Tris-HCl, pH 8.0), crushed with a French press and ultrasonicated for three turns. Subsequently, the supernatants were separated by centrifugation at 12,000 *g* for 30 min at 4°C and then loaded into Ni affinity chromatography column (GE Healthcare, Chicago, IL, United States). After being washed by 20 column volumes of lysis buffer containing 30 mM imidazole, the purified protein was eluted from Ni affinity resin by elution buffer (300 mM imidazole and 500 mM NaCl in 20 mM Tris-HCl, pH 8.0).

### Sortase A–Mediated Ligation Reaction

The sortase A–mediated ligation reaction was in ligation buffer containing 50 mM Tris-HCl, pH 7.0, 150 mM NaCl, and 20 mM CaCl_2_. The ligation was conducted with 20 μM RecQ^516^-LPETG, 100 μM Cy5-labeled GGG-HRDC, and 50 μM sortase A at 34°C for 1 h.

### RecQ Reaction Buffer

RecQ reaction buffer contains 2 mM MgCl_2_, 0.1 mg/mL bovine serum albumin, and 50 mM KCl in 20 mM Tris-HCl, pH 7.5. For single-molecule measurements, 0.8% D-glucose, 1 mg/mL glucose oxidase (266,600 units/g, Sigma–Aldrich, St. Louis, MO, United States), 0.4 mg/mL catalase (2,000–5,000 units/mg, Sigma–Aldrich), and 1 mM Trolox (Sigma–Aldrich) were added to the reaction buffer (Roy et al., 2008).

### Single-Molecule Fluorescence Data Acquisition

All oligonucleotides required to make DNA substrates were purchased from Sangon Biotech. DNA constructs used in single-molecule measurements were carried out as described previously (Wang et al., 2019), and the sequences were listed in Supplementary Table S2. SmFRET study was carried out with a home-built objective-type total-internal-reflection microscopy as described previously (Roy et al., 2008; Wang et al., 2019). Cy3 was excited by a 532-nm Sapphire laser (Coherent, Santa Clara, CA, United States). An oil immersion objective (100×, N.A.1.49) was used to generate an evanescent field of illumination. Fluorescence signals from Cy3 and Cy5 were split by a dichroic mirror and finally collected by an electron-multiplying charge-coupled device camera (iXON; Andor Technology, South Windsor, CT, United States). Fluorescence imaging processes were controlled and recorded by MetaMorph (Molecular Device, Sunnyvale, CA, United States). The coverslips (Fisher Scientific, Pittsburgh, PA, United States) and slides were cleaned thoroughly by a mixture of sulfuric acid and hydrogen peroxide, acetone, and sodium ethoxide, and then the surfaces of coverslip were coated with a mixture of 99% mPEG (m-PEG-5000, Laysan Bio, Inc., Arab, AL, United States) and 1% of biotin-PEG (biotin-PEG-5000, Laysan Bio, Inc.). Streptavidin (10 μg/mL) was added to the microfluidic chamber made of the PEG-coated coverslip and incubated for 10 min. After washing, 100 pM DNA was immobilized for 10 min. Then free DNA was removed by washing with the reaction buffer. We used an exposure time of 100 ms for all single-molecule measurements at a constant temperature of 22°C. To obtain the fraction of DNA unwinding vs. time, a series of movies were recorded with 1-s duration at indicated times, and the Cy3 spots were counted to represent the number of remaining DNA molecules.

### FRET Data Analyses

The FRET efficiency was calculated using *I*_*A*_/(*I*_*D*_ + *I*_*A*_), where *I*_*D*_ and *I*_*A*_ represent the intensity of donor and acceptor, respectively. The leakage from Cy3 Channel to Cy5 Channel is about 10%; therefore, we deducted the leakage when exporting the single-molecule fluorescence intensity by the software “smCamera” (Roy et al., 2008). Basic data analysis was carried out by scripts written in MATLAB, and all data fitting was generated by Origin 8.0. An automated step-finding method (from http://bio.physics.illinois.edu/HaMMy.asp) was employed to characterize the association and dissociation of RecQ, and the E_FRET_ value and dwell time (*t*) for each reaction were determined accordingly. The resulting histograms of FRET values and dwell time from more than 150 molecules were fitted with multipeak Gaussian distribution or Gamma distribution or single-exponential decay, respectively.

## Results

### Labeling the HRDC Domain With a Single Cy5 Fluorophore Mediated by Sortase A Ligation

Maleimide-activated fluorophores are monoreactive dyes that can selectively couple with cysteines in peptides or proteins to generate specifically labeled conjugates and have been widely used in enzymology experiments. As full-length RecQ contains 11 cysteine residues, it is difficult to label the HRDC domain of RecQ with a single fluorophore directly. To avoid non-specific fluorescent labeling, based on the flexible linker (∼22 aa) between the RQC and HRDC domains, we established a scheme (Figure 1B) to specifically label the HRDC domain with a single Cy5-maleimide fluorophore mediated by sortase A, which could fuse an LPXTG recognition motif to an N-terminal GGG-containing motif, thus regenerating a native amide bond and a recombinant protein.

**FIGURE 2:**
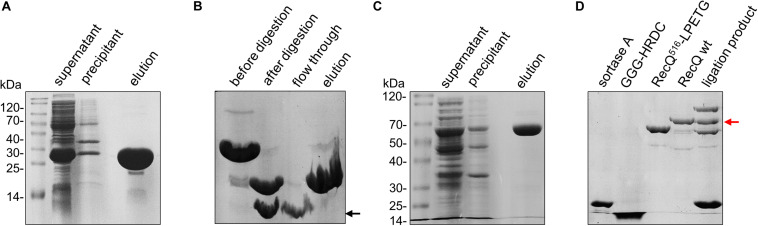
Purification of protein constructs. **(A)** Expression and purification of sumo-GGG-HRDC (∼21 kDa) by Ni affinity resin and analysis by 15% SDS-PAGE. **(B)** Purification of Cy5 labeled GGG-HRDC (∼9 kDa) analyzed by 15% SDS-PAGE. Cy5-labeled sumo-GGG-HRDC was digested using SUMO protease, and then Cy5-labeled GGG-HRDC was purified by Ni affinity resin. The target protein is indicated by the black arrow. **(C)** Expression and purification of RecQ^516^-LPETG (∼59 kDa) by Ni affinity resin and analysis by 10% SDS-PAGE. **(D)** The ligation of Cy5-labeled GGG-HRDC with RecQ^516^-LPETG analyzed by 10% SDS-PAGE; the excess free Cy5-labeled GGG-HRDC in the ligation product was removed by Ni affinity resin. The target protein is indicated by the red arrow.

First, owing to the absence of cysteine in the HRDC domain, E610C was designed to label the Cy5-maleimide fluorophore. To obtain sortase A recognition sequence H_2_N-GGG, we conducted overlap PCR to fuse HRDC (amino acids 524–610) with a SUMO tag (12 kDa) and amino acid sequence “GGG” at the N-terminal (Figure 1B). The recombinant sequence was cloned into pET15b and expressed in BL21 (DE3). Then, the recombinant protein sumo-GGG-HRDC (∼21 kDa) was purified by Ni affinity chromatography (Figure 2A). After that, sumo-GGG-HRDC was labeled with a 15-fold molar excess of Cy5-maleimide fluorophore at 20°C for 1 h, and the free Cy5 dye was removed by Ni affinity chromatography. Then, the Cy5-labeled sample was digested with SUMO protease at 4°C overnight (Figure 2B). Finally, the Cy5-labeled GGG-HRDC (∼9 kDa) was purified again by Ni affinity chromatography (indicated by black arrow in Figure 2B). We then used the preset-program “Protein and Labels” of Thermo Scientific Nanodrop 2000c to measure the concentration of protein and Cy5. As the Cy5 labeling was single and site-specific, and the free Cy5 dye was removed, the labeling efficiency was calculated by the ratio of the Cy5 concentration to the protein concentration, and it was more than 95%.

In the meantime, we conducted overlap PCR to fuse sortase A recognition sequence “LPETG” at the C-terminal of RecQ core (amino acids 1–516, designated as RecQ^516^-LPETG, ∼59 kDa). RecQ^516^-LPETG was cloned into pET15b and expressed in BL21 (DE3). Similarly, RecQ^516^-LPETG was purified by Ni affinity chromatography (Figure 2C). Additionally, the expression and purification of sortase A (∼19 kDa) were carried out as described previously (Mao et al., 2004).

The sortase A–mediated ligation reaction was conducted with 20 μM RecQ^516^-LPETG, 100 μM Cy5-labeled GGG-HRDC, and 50 μM sortase A in ligation buffer (50 mM Tris-HCl, pH 7.0, 150 mM NaCl, and 20 mM CaCl_2_) at 34°C for 1 h. At last, Ni affinity chromatography was used again to remove the excess free Cy5-labeled GGG-HRDC, as free Cy5-labeled GGG-HRDC will disturb the fluorescence signal; the ligation efficiency was ∼30%, as determined by sodium dodecyl sulfate–polyacrylamide gel electrophoresis (SDS-PAGE) (Figure 2D). Therefore, we succeeded in labeling the HRDC domain of *E. coli* RecQ with a single Cy5 fluorophore, and the Cy5-labeled recombinational RecQ was designated as RecQ-Cy5.

### Cy5-Labeled RecQ Exhibits the Same Unwinding Activities as Those of Wild-Type RecQ

**FIGURE 3:**
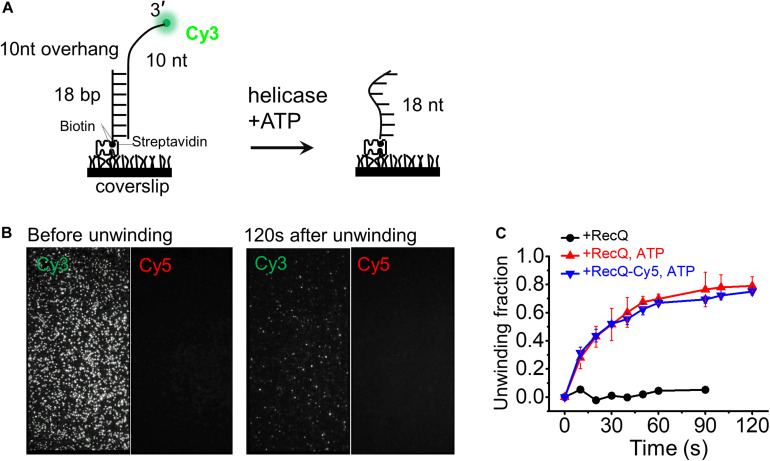
Cy5-labeled RecQ exhibits the same dsDNA unwinding activity as that of wild-type RecQ. **(A)** Schematic representation of the smFRET experiment DNA (10-nt overhang). DNA was surface immobilized using a biotin–streptavidin bridge onto a polyethylene glycol passivated coverslip. Cy3 signal is lost when Cy3-labeled 3′ overhang strand is unwound by RecQ. **(B)** Representative field of view before and after unwinding by 5 nM RecQ-Cy5 and 2 mM ATP. Each white dot represents fluorescent DNA molecules. **(C)** Fractions of unwound DNA molecules on coverslip vs. time in indicated reaction conditions. Error bars represent standard deviation (SD) from three experiments.

First, we designed the 10-nt overhang DNA substrate to evaluate the activity of RecQ-Cy5. The DNA substrate contained a Cy3 fluorophore labeled at the 3′ end of the 10-nt overhang, and a biotin labeled at the 3′ end of the complementary strand to fix the DNA substrate on the surface of the reaction chamber, such that the Cy3 signal would disappear promptly if the 18-bp dsDNA was fully unwound (Figure 3A).

Then, we measured the unwinding activity and unwinding fractions of the 18-bp 10-nt DNA by 5 nM RecQ-Cy5 or wild-type RecQ using 2 mM ATP. To obtain the fraction of DNA unwinding vs. time, the fluorescence is intermittent excited, and a series of movies were recorded with 1-s duration at indicated times, and the Cy3 spots were counted to represent the number of remaining DNA molecules, as previously reported (Wang et al., 2018). We found that RecQ-Cy5 could unwind dsDNA (Figure 3B), and the unwinding fractions of 18-bp 10-nt DNA by RecQ-Cy5 and wild-type RecQ had little difference (Figure 3C). Therefore, we concluded that Cy5 labeling or sortase A ligation did not affect the RecQ-Cy5 activity, and RecQ-Cy5 occupied the same unwinding activities as wild-type RecQ.

### The HRDC Domain Can Directly Interact With RecA Domains Repeatedly

**FIGURE 4:**
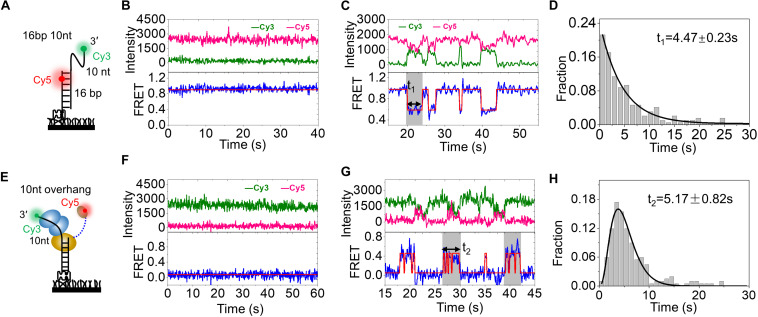
HRDC domain can directly interact with RecA domains repeatedly. **(A)** Schematic representation of the smFRET experimental DNA (16 bp 10 nt). **(B)** The typical fluorescence intensity trace (upper panel) and corresponding smFRET-time traces (lower panel) of 16 bp 10 nt. **(C)** The typical fluorescence intensity trace (upper panel) and corresponding smFRET-time traces (lower panel) of 16 bp 10 nt in the presence of 5 nM wild-type RecQ. The dwell time (*t*_1_) of each RecQ is exhibited by the gray box. An automated step-finding algorithm was used to identify the FRET states (red line in the lower panel). **(D)** Distribution of dwell time (*t*_1_) collected from ∼150 molecules and single exponential fitting with an average time of 4.47 ± 0.23 s by 5 nM RecQ. **(E)** Schematic representation of the smFRET experiment DNA (10-nt overhang). **(F)** The typical fluorescence intensity trace (upper panel) and corresponding FRET traces (lower panel) of smFRET experimental DNA, 10-nt overhang. **(G)** The typical fluorescence intensity trace (upper panel) and corresponding smFRET-time traces (lower panel) of 10-nt overhang in the presence of RecQ-Cy5. The dwell time (*t*_2_) of RecQ-Cy5 is exhibited by the gray box. **(H)** Distribution of dwell time (*t*_2_) collected from ∼150 molecules and gamma distribution fitting with an average time of 5.17 ± 0.82 s by RecQ-Cy5.

Before detecting the dynamic trajectory of Cy5-labeled HRDC, we first detected the binding process of wild-type RecQ by smFRET. The DNA substrate 16 bp with 3′ 10-nt overhang was prepared (Figure 4A). The donor (Cy3) was labeled at the 3′ end of the 10-nt overhang, and the acceptor (Cy5) was labeled at the 4th nucleotide from the 5′ end in the 16-nt stem strand so that a relatively high FRET efficiency (E_FRET_) of ∼0.92 was detected because of the high flexibility of ssDNA (Figure 4B and Supplementary Figure S1A). Upon addition of wild-type RecQ, smFRET-time traces exhibited periodic fluctuations between an E_FRET_ of ∼0.92 and ∼0.54 (Figure 4C and Supplementary Figure S1A), as RecQ-binding stretched ssDNA. Then, we analyzed the dwell time of the periodic fluctuations, which has been indicated by the gray box and represents RecQ bound to the DNA. The dwell time *t*_1_ collected from more than 150 molecules and followed single-exponential decay with an average time of 4.47 ± 0.23 s (Figure 4D).

We then detected the dynamic trajectory of RecQ-Cy5 binding to the 10-nt overhang DNA substrate, in which the donor (Cy3) was labeled at the 3′ end of the overhang (Figure 4E). The FRET efficiency of the substrate was ∼0 because of the lack of the acceptor (Cy5) (Figure 4F and Supplementary Figure S2A). After allowing RecQ-Cy5 (5 nM) binding to substrate DNA for 2 min, we washed off the free protein in the reaction chamber using reaction buffer and recorded the binding process. The binding process exhibited rapid periodic fluctuations between *E*_FRET_ of ∼0 and ∼0.5 within a certain period, as indicated by the gray box in Figure 4G. The total duration time of each rapid periodic fluctuation (*t*_2_ in Figure 4G) was counted, and it followed the gamma distribution (Zhou et al., 2014; Wu et al., 2015) with an average time of 5.17 ± 0.82 s (Figure 4H), which is comparable to the dwell time (*t*_1_ = 4.47 ± 0.23 s) when wild-type RecQ was binding to the 3′ overhang DNA, as shown in Figure 4C; this result indicated that the rapid periodic fluctuations in *t*_2_ were caused by HRDC rapid periodic association to and dissociation from RecA domains after the binding of RecQ-Cy5 to substrate DNA, rather than RecQ repeatedly binding to DNA.

We constructed a rough structure of *E. coli* RecQ in complex with a partial duplex according to the existing structural data (Supplementary Figure S2B). The simulated structure presented that RecA domain of RecQ would bind with ∼9-nt ssDNA, and HRDC domain may directly interact with RecA domain. Meanwhile, the C-terminal of HRDC domain from RecQ was just ∼6.2 nM away from the 10th base of the 3′ overhang DNA (Supplementary Figure S2B), which could exactly give rise to a FRET at ∼0.45. Therefore, under our experimental conditions, the rapid periodic fluctuations of FRET between ∼0 and ∼0.49, as indicated by the gray box in Figure 4G, could be caused by the interaction of the Cy5-labeled HRDC domain with RecA domains repeatedly after RecQ binding to DNA. Therefore, we concluded that the site-specific fluorescent labeling method established by us could visualize the interaction between molecules, and HRDC would repeatedly interact with RecA domains when RecQ binds to 3′ overhang DNA.

### The HRDC Domain Can Directly Interact With the 3′ and 5′ Overhang DNA Repeatedly

**FIGURE 5:**
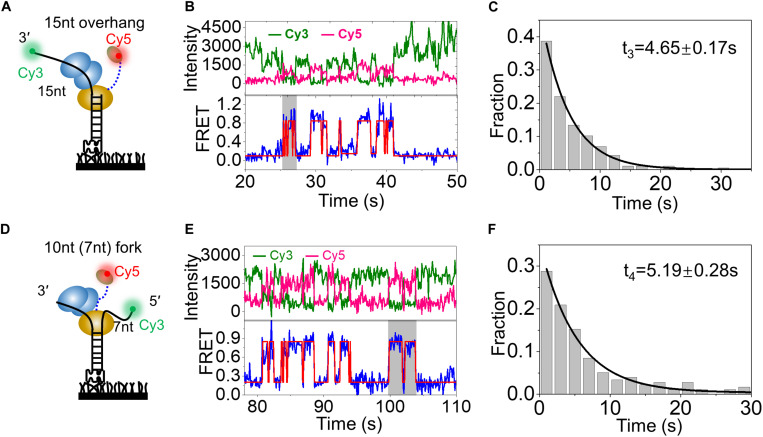
HRDC domain can directly interact with 3′ and 5′ overhang DNAs repeatedly. **(A)** Schematic representation of 15-nt overhang DNA. **(B)** The typical fluorescence intensity trace (upper panel) and corresponding smFRET-time traces (lower panel) of 15-nt overhang in the presence of RecQ-Cy5. **(C)** Distribution of dwell time (*t*_3_) collected from ∼150 molecules and single exponential fitting with an average time of 4.65 ± 0.17 s. **(D)** Schematic representation of 10 nt (7 nt) fork DNA. **(E)** The typical fluorescence intensity trace (upper panel) and corresponding smFRET-time traces (lower panel) of 10-nt (7 nt) fork in the presence of RecQ-Cy5. The dwell time (*t*_4_) of RecQ-Cy5 is exhibited by the gray box. **(F)** Distribution of dwell time (*t*_4_) collected from ∼150 molecules and followed with Gaussian distribution fitting with an average time of 5.19 ± 0.28 s by RecQ-Cy5.

Then, a substrate DNA with 15-nt 3′ overhang was designed, and the donor (Cy3) was also labeled at the 3′ end of the overhang (Figure 5A). After RecQ-Cy5 (5 nM) binding to substrate DNA for 2 min and when the free protein was washed off by reaction buffer, FRET rapidly increased from ∼0 to ∼0.84 repeatedly, as indicated in the gray box in Figure 5B (Figure 5B and Supplementary Figure S3A); this was much higher than that of 10-nt 3′ overhang DNA, corresponding to RecQ binding to substrate DNA, and Cy5-labeled HRDC domain much closer to the 3′ end of the 15-nt overhang than the 10-nt overhang DNA. The average duration of rapid periodic fluctuation (*t*_3_) was counted from more than 150 molecules and followed by single exponential decay fitting with an average time of 4.65 ± 0.17 s (Figure 5C), which was also comparable to the dwell time when wild-type RecQ was binding to 3′ overhang DNA (Figure 4D). These results indicate that when there is additional ssDNA (more than 10 nt) in the 3′ end of the overhang, the Cy5-labeled HRDC domain can rapidly and repeatedly bind to the 3′ end of the overhang directly.

In the meantime, a fork DNA with 10-nt 3′ overhang and 7-nt 5′ overhang was designed (Figure 5D), and the donor (Cy3) was labeled at the 5′ end of the overhang. Under the same conditions, FRET also rapidly increased from ∼0 to ∼0.92 repeatedly (Figure 5E and Supplementary Figure S3B), thereby indicating that Cy5 was almost completely close to Cy3. The average duration of rapid periodic fluctuation (*t*_4_) was also determined. *t*_4_ also followed single exponential decay fitting with an average duration of 5.19 ± 0.28 s (Figure 5F). The aforementioned results indicated that the Cy5-labeled HRDC domain could also bind to the 5′ end of overhang repeatedly after RecQ binding to the substrate DNA. Based on these data, we concluded that HRDC could directly interact with 3′ and 5′ overhang DNA repeatedly after RecQ binding to the substrate DNA.

## Discussion

Fluorescent dyes, such as cyanine and Alexa Fluor, have been widely used in *in vitro* enzymology experiments because they are easy to label and have little effect on enzymatic activity (Xi and Deprez, 2010). NHS ester-activated and maleimide-activated dyes have been widely adopted and can be specifically coupled with the amine group of lysine residues or the N-terminus and the sulfhydryl of cysteine residues, respectively. However, as lysine residues are more abundant in proteins (average abundance, 5.9%) and are frequently involved in binding interactions (Rosen and Francis, 2017), cysteine residues (average abundance, 1.9%) always stand out as uniquely reactive sites for labeling fluorescent dyes to detect conformational changes or trajectory of proteins in smFRET experiments (Christian et al., 2009; Hou et al., 2015; Bell and Kowalczykowski, 2016). Usually, to label one fluorophore to a definite cysteine site, other cysteine residues must be mutated. Meanwhile, it should ensure that the structure and activity of the protein will not be affected when mutating cysteine residues. Therefore, fluorophore labeling of proteins is a key limiting factor in many smFRET experiments.

*E. coli* RecQ contains 11 cysteine residues, and it is difficult to label the HRDC domain with fluorophore directly as it is almost impossible to mutate all cysteines without affecting the structure and activity of RecQ (Ren et al., 2008). Therefore, we established a scheme for specifically labeling HRDC with a single Cy5-maleimide fluorophore, which is mediated by sortase A and is dependent on the flexible linker (∼22 aa) between the RQC and HRDC domains (Figure 1).

Sortase A is an efficient and versatile tool for protein modification, which can fuse an LPXTG recognition motif to an N-terminal GGG-containing motif, thereby regenerating a native amide bond (Li et al., 2020). Therefore, our scheme is mainly appropriate for proteins that contain multiple or immutable cysteines on the non-labeled domain, and there is a non-functional flexible linker between the labeled domain and non-labeled domain for recognition and ligation by sortase A. Meanwhile, it is worth noting that our scheme replaces only six non-functional amino acids on the flexible linker and will not inlet extra fluorescent domain or peptide fragment, which is highly likely to affect the protein activity or structure, thereby leading to perturbations in the real-time detection system because of the high sensitivity of smFRET.

The ligation efficiency of sortase A is affected by many factors, such as the steric hindrance of the proligation motifs, ligation buffer, and temperature (Broguiere et al., 2018). Usually, a low-salt solution (150 mM Na^+^), appropriate Ca^2+^ concentration (5–60 mM), and proper temperature (∼37°C) can increase the ligation efficiency of sortase A (Dasgupta et al., 2011; Levary et al., 2011; Warden-Rothman et al., 2013; Broguiere et al., 2018). Meanwhile, it should be noted that low salt solution and high temperature may cause protein denaturation during ligation. Certainly, apart from sortase A–mediated ligation, other ligation methods may also be feasible, such as protein trans-splicing by split inteins (Friedel et al., 2019; Yao et al., 2020).

We visualized that Cy5-labeled HRDC domain could repeatedly interact with RecA domains and bind to 3′ and 5′ overhang DNAs repeatedly after RecQ binding to DNA for the first time (Figures 4, 5). Repeated interaction of HRDC with RecA domains may inhibit ATP binding or ATP hydrolysis, thereby affecting ADP, and/or Pi release by RecA, and thus slowing down the ssDNA translocation and dsDNA unwinding processes, as previously reported (Chatterjee et al., 2014; Kocsis et al., 2014; Harami et al., 2015). The HRDC domain directly binds to the overhang DNA and may lead to multiple functions. While binding to the 3′ overhang DNA, HRDC may directly slow down the ssDNA translocation or dsDNA unwinding, as previously reported (Kocsis et al., 2014; Harami et al., 2015). Meanwhile, by binding to 5′ overhang DNA, HRDC may induce pausing and shuttling during unwinding dsDNA (Harami et al., 2017). Furthermore, we have also discovered that HRDC can facilitate strand-switch process and restrain RecQ patrolling on 5′ ssDNA after HRDC establishes contact with the 5′ overhang DNA (Teng et al., in preparation).

Owing to their low sequence identity and highly different surface properties, the HRDC domain from different RecQ family helicases may exhibit differentiated functions, especially the DNA binding and protein–protein interaction activities (Brosh, 2013), which may need to be detected at the single-molecule level in future studies. Our research not only directly reveals the functional mechanism of the HRDC domain during *E. coli* RecQ transaction with different DNA during DNA repair and DNA recombination, but also provides a feasible method for site-specific labeling of a domain with a single fluorophore and thus facilitates the visualization of molecular interactions, conformational changes in proteins, enzymatic activity, and individual protein moving in real time at the single-molecule level.

## Data Availability Statement

The raw data supporting the conclusions of this article will be made available by the authors, without undue reservation, to any qualified researcher.

## Author Contributions

X-GX and YX conceived and supervised the study. X-GX and F-YT designed experiments. F-YT, Z-ZJ, and L-YH performed experiments. F-YT and MG analyzed data. F-YT, Z-ZJ, and X-MH wrote the manuscript. X-GX, YX, and FC made manuscript revisions. All authors contributed to the article and approved the submitted version.

## Conflict of Interest

The authors declare that the research was conducted in the absence of any commercial or financial relationships that could be construed as a potential conflict of interest.
