# Supplementary material for: A Toolbox for Site-Specific Labeling of RecQ Helicase With a Single Fluorophore Used in the Single-Molecule Assay
Source: Front Mol Biosci. 2020 Sep 25;7:586450. doi: 10.3389/fmolb.2020.586450 (PMC7545742; doi:10.3389/fmolb.2020.586450)
Supplement: Supplementary file 1 [file Data_Sheet_1.pdf]

Supplementary Material

A toolbox for site-specific labeling of RecQ helicase with a single fluorophore used in the single-molecule assay

Fang-Yuan Teng[1,2,3,4,†], Zong-Zhe Jiang[1,3,†], Ling-Yun Huang[2], Man Guo[3], Feng Chen[1], Xi-Miao Hou[2], Xu-Guang Xi[2,5,*] , and Yong Xu[1,3,*]

[1] Experimental Medicine Center, The Affiliated Hospital of Southwest Medical University, Luzhou, Sichuan 646000, China

[2] State Key Laboratory of Crop Stress Biology for Arid Areas and College of Life Sciences, Northwest A&F University, Yangling, Shaanxi 712100, China

[3] Department of Endocrinology and Metabolism, and Cardiovascular and Metabolic Diseases Key Laboratory of Luzhou, and Sichuan Clinical Research Center for Nephropathy, The Affiliated Hospital of Southwest Medical University, Luzhou, Sichuan 646000, China

[4] Academician (Expert) Workstation of Sichuan Province, The Affiliated Hospital of Southwest Medical University, Luzhou, Sichuan 646000, China

[5] LBPA, Ecole normale supérieure Paris-Saclay, CNRS, Université Paris Saclay, 61, avenue du Président Wilson, F-94235 Cachan, France

*Correspondence: Xu-Guang Xi, *E-mail*: xxi01@ens-cachan.fr, And Yong Xu, *E-mail*: xywyll@aliyun.com

† These authors have contributed equally to this work.

Supplementary Tables

Table S1. DNA primers used in protein constructs (5′-3′)

Name	**Sequence**
Sumo-F	CGGGAATTCCATATGAGCGATAGCGAAGTGAACC
Sumo-R	ATAGTTGCCGCCGAACGATTTCTGGCCACCACCGCCGCCAATCTGTTCGCGATGCGCT
HRDC-F	AGCGCATCGCGAACAGATTGGCGGCGGTGGTGGCCAGAAATCGTTCGGCGGCAACTAT
HRDC-R	CCCTCGAGTTAGTCGACGCATTCGTCATCGCCATCAACATGCGCA
$RecQ^{516}$-LPETG-F	CGGGAATTCCATATGAATGTGGCGCAGGCGGAAGTGTTGA
$RecQ^{516}$-LPETG-R	GCCCTCGAGTTACGCGCCAGTTTCAGGGAGCACGATACGCGGCACGGCAAGTTGCA

Table S2. Sequences of substrates used in the smFRET experiments (5′-3′)

	**Substrates for single-molecule FRET**
16bp 10nt	AATCCGTAGAGCAGAGGTGTGTGTGG-Cy3
Stem	CTCT(iCy5)GCTCGACGGATT-Biotin
10nt overhang	AATCCGTAGAGCAGAGGTGTGTGTGGTG-Cy3
Stem	ACCTCTGCTCGACGGATT-Biotin
15nt overhang	AATCCGTAGAGCAGAGGTGTGTGTGGTGTTTTT-Cy3
Stem	ACCTCTGCTCGACGGATT-Biotin
10nt (7nt) fork	Cy3-TTTTTTTATGTATGACAAGGAAGG
Stem	biotin-CCTTCCTTGTCATACATAAATTTTTTT

Supplementary Figures

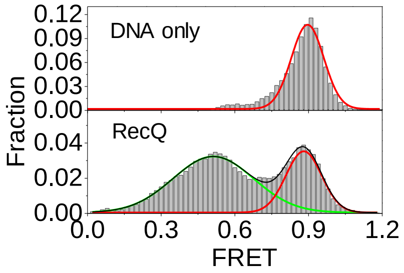

Figure S1. Distribution of the average EFRET of 16bp 10nt DNA before and after adding wild-type RecQ. The distribution was collected from ~150 molecules, Single-peak Gaussian fitting gave a peak for DNA at 0.92, and multi-peak Gaussian fitting gave two peaks at 0.92 and 0.54 after adding wild-type RecQ.

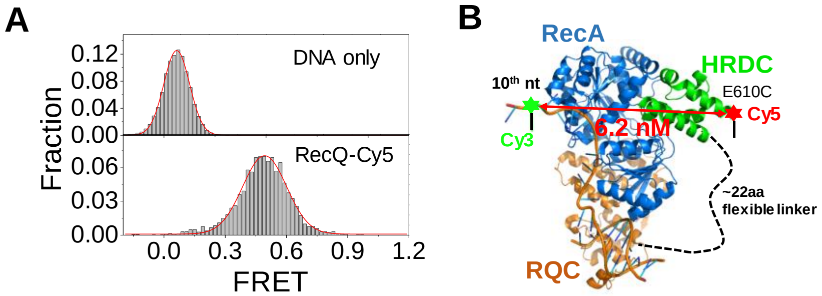

Figure S2. Distribution of the average EFRET of 10nt overhang DNA before and after adding Cy5-RecQ. (A) Distributions of the average E_{FRET} collected from ~150 molecules. Single-peak Gaussian fitting gave a peak for DNA at ~0, and for RecQ-Cy5 at ~0.49. The FRET distribution after adding RecQ-Cy5 was counted from the reaction part in the grey box. **(B)** A simulated structure of *E. coli* RecQ in complex with partial duplex, depending on the structure data of *E. coli* RecQ (PDBid 1OYW), HRDC (1WUD), *Cs*RecQ (4TMU) and human BLM (4O3M and 4CGZ). It was indicated that the C-terminal of HRDC domain from RecQ was just ~6.2 nM away from 10th base of 3′ the overhang DNA, which could exactly give rise to a FRET at ~0.45.

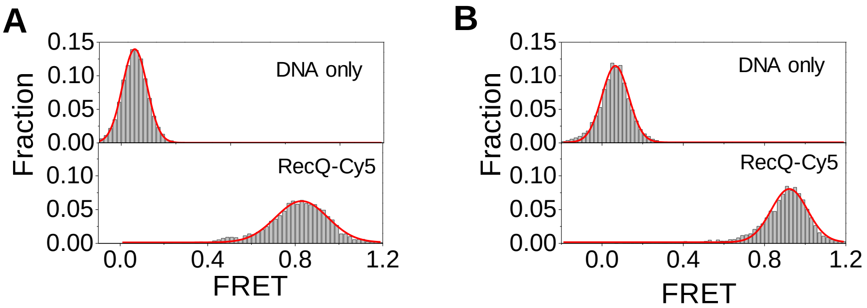

Figure S3. Distribution of the average EFRET of 15nt overhang DNA and 10nt (7nt) fork DNA. (A) Distributions of the average E_{FRET} of 15nt overhang DNA collected from ~150 molecules. Single-peak Gaussian fitting gave a peak for DNA at ~0, and for RecQ-Cy5 at ~0.84. The FRET distribution was counted from the reaction part in the grey box. **(B)** Distributions of the average E_{FRET} of 10nt (7nt) fork DNA collected from ~150 molecules. Single-peak Gaussian fitting gave a peak for DNA at ~0, and for RecQ-Cy5 at ~0.9.
